# Novel Prognostic and Predictive miRNA Biomarkers Shape the Landscape of T Cell Dysfunction in Cancer

**DOI:** 10.1111/jcmm.71117

**Published:** 2026-04-02

**Authors:** Hong‐jiu Wang, Xiao‐ling Wen, Zhe‐yu Wu, Na Wang, Shu‐heng Fu, Fei‐fan Xiong, Jiang‐ying Liang, Deng‐hui Guo, Si‐rui Li, Jie Shen, Xiao‐ling Gao, Zhen‐zhen Wang

**Affiliations:** ^1^ School of Intelligent Medicine and Technology, Big Data Research Center Hainan Medical University Haikou China; ^2^ College of Bioinformatics Science and Technology Harbin Medical University Harbin China; ^3^ Shanghai Jiao Tong University Shanghai China; ^4^ The Medical Laboratory Center Hainan General Hospital Haikou China; ^5^ Key Laboratory of Reproductive Health Diseases Research and Translation (Hainan Medical University), Ministry of Education The First Affiliated Hospital of Hainan Medical University, Hainan Medical University Haikou Hainan China

**Keywords:** cancer subtype, immunocompetence, miRNA, pan‐cancer, prognosis biomarker, T cell dysfunction, tumour microenvironment

## Abstract

T cell dysfunction (TCD) plays a critical role in cancer progression and significantly impacts patient outcomes. Despite its importance, the exact molecular mechanisms underlying TCD remain poorly understood. To address this, we constructed a comprehensive pan‐cancer landscape of TCD, with a particular focus on identifying miRNA biomarkers that define and predict TCD severity. Our analysis revealed six key miRNAs (miR‐203b, miR‐214, miR‐4772, miR‐141, miR‐200a, and miR‐200b) that were closely associated with varying degrees of TCD. These prognostic miRNAs not only exhibited distinct expression patterns across four identified TCD subtypes (from low to high TCD severity) but also demonstrated strong predictive performance in classifying patients with different levels of TCD. The identified miRNA signatures serve as reliable biomarkers for stratifying patients into high‐risk and low‐risk groups, with higher TCD levels correlating to poorer overall survival. In addition to miRNA biomarkers, we observed that patients with severe TCD exhibited increased infiltration of immune cells and macrophages and dysregulation of DNA methylation patterns. Patients with higher degrees of TCD displayed low methylation levels, which further contributed to the progression of T cell dysfunction. In summary, our study highlights the pivotal role of miRNA biomarkers in shaping the landscape of T cell dysfunction across cancers. These miRNAs serve as both prognostic indicators and predictive tools, enabling accurate classification of TCD severity and offering new avenues for therapeutic exploration and patient stratification in cancer immunotherapy.

## 1 Introduction

In recent years, there has been a significant surge in research aimed at elucidating the impairments of T cells within the intricate landscape of cancer. This comprehensive exploration encompasses a spectrum of states, including exhaustion, senescence, exclusion, and dysfunction, each contributing to the nuanced mechanisms by which cancer cells evade immune surveillance and foster tumour progression [1, 2, 3].

Understanding the diverse manifestations of TCD within the context of cancer is imperative for the development of efficacious T cell‐centered therapies [4, 5]. T cell exhaustion epitomizes a state characterized by the sustained expression of inhibitory receptors, distinct alterations in transcriptional profiles, and compromised effector function. This phenomenon profoundly undermines the immune system's ability to combat infections and malignancies, notably evidenced in the cytolytic immune activity within primary non‐small cell lung cancer (NSCLC) [6, 7]. T cell senescence delineates a gradual decrement in cellular proliferation, differentiation, and overall physiological functionality during the aging process, as discerned through the identification of ten molecular markers [8]. Moreover, the interplay between T cell exclusion and dysfunction intricately correlates with the levels of cytotoxic T lymphocytes (CTLs). Tumoral immune evasion via T cell exclusion arises from the presence of immunosuppressive factors coupled with minimal CTL infiltration, thereby shielding immunogenic tissues from immune surveillance [9]. Conversely, heightened CTL levels often precipitate TCD [9], further illustrating the nuanced dynamics within the tumour microenvironment. Numerous mechanistic pathways collectively contribute to TCD in solid tumours [10], orchestrated by a plethora of inhibitory signals emanating from the intricate tumour microenvironment [11, 12]. Comprehending these distinct states of TCD holds pivotal significance in the pursuit of devising efficacious T cell‐oriented therapeutic interventions.

Despite recent progress in understanding T cell dysfunction within the context of cancer, significant challenges persist. The complex interplay among different manifestations of TCD, encompassing exhaustion, senescence, exclusion, and general dysfunction, remains poorly elucidated. Additionally, the absence of definitive markers for defining and characterizing TCD poses a substantial hurdle. Moreover, the intricate molecular mechanisms driving TCD across various cancer types are still not fully delineated. Consequently, there is a pressing need for the development of effective strategies to overcome these limitations and advance our understanding of TCD in cancer.

Our study aims to provide a comprehensive portrayal of TCD spanning diverse cancer types, delineating four discernible subtypes marked by a progressive exacerbation of T cell dysfunction. Additionally, we delve into the clinical relevance of these subtypes and their impact on the immune microenvironment. Furthermore, we have identified miRNA biomarkers associated with TCD, enabling the prediction of varying degrees of dysfunction in patients and enhancing prognostic evaluations. In summary, our investigation furnishes an exhaustive examination of TCD, furnishing invaluable insights for forthcoming cancer investigations and therapeutic endeavours.

## 2 Materials & Methods

### 2.1 Microarray Data Collection

Level 3 RNA‐seq gene expression datasets were downloaded from The Cancer Genome Atlas [13] (TCGA) for 33 solid tumour types, encompassing RNA‐Seq (FPKM) data for 60,483 genes across 11,093 samples. The dataset included 730 normal samples and 10,363 disease samples. Expression data for 1881 miRNAs were also obtained for 10,813 samples. Clinical information for all 11,093 samples was retrieved from the TCGA Pan‐Cancer Clinical Data Resource (TCGA‐CDR).

To validate the findings, miRNA expression data and corresponding clinical information were acquired from the Gene Expression Omnibus (GEO) database. Two datasets were utilized: GSE92928, which contains data from 165 colorectal cancer (CRC) samples profiled for non‐coding RNA, and GSE73581, which comprises data from 179 epithelial ovarian cancer (EOC) samples profiled for non‐coding RNA.

Additionally, pan‐cancer DNA methylation data generated using the 450 K platform were retrieved from UCSC Xena. DNA methylation values (β values) for each array probe were analysed, with values ranging from 0 to 1 to indicate the degree of methylation. Higher β values represented hypermethylation, while lower β values indicated hypomethylation.

### 2.2 T Cell Dysfunction Signature Genes

The signature genes for 26 T cell exhaustion‐related genes and 25 T cell senescence related genes were obtained from the published paper [14]. The signature genes for 292 T cell exclusion‐associated genes and 90 TCD related genes were obtained from the published paper [9].

### 2.3 Immune Cell Infiltration and Other Signature Genes Data

Immune cell infiltration data related to the tumour microenvironment were obtained from the TIMER database. Fifty genes associated with immune checkpoints (Table S1) were sourced from Sino Biological (https://www.sinobiological.com/category/immune‐checkpoint‐proteins‐elite). The 160 genes associated with T cell inflammation (Table S2) were obtained from the article [15]. Additionally, 1244 genes related to 14 cell states were obtained from CancerSEA [16] (http://biocc. hrbmu.edu.cn/CancerSEA/home.jsp). A total of 1042 target genes of miRNAs were identified using the miRCarta [17] database (https://mircarta.cs.uni‐saarland.de/).

### 2.4 Construction of the T Cell Dysfunction Profile

A dysfunction profile of T cells was constructed based on the TCD signature genes and their expression profiles in normal and disease samples from the TCGA dataset. The profile is represented as a matrix, where the rows correspond to four TCD states, the columns represent disease samples, and the values in the matrix represent *p*‐values obtained from a hypergeometric test.

(1) $$P\left(k,N,M,n\right)=\frac{\left(\begin{array}{c} M\\ k \end{array}\right)*\left(\begin{array}{c} N-M\\ n-k \end{array}\right)}{\left(\begin{array}{c} N\\ n \end{array}\right)}$$

N is the number of all genes, M is the number of differentially expressed genes (DEGs) based on fold change values (FC > 2 or FC < 0.5) that equal dividing the expression of disease samples by the average expression of normal samples, n is the number of signature genes in each TCD state, k is the number of DEGs (FC > 2 or FC < 0.5) in each T cell dysfunction state.

### 2.5 Identification of Four T Cell Dysfunction Subtypes

By applying the ConsensusClusterPlus [18] R package with specific parameter settings, namely a maximum number of clusters (max k) set to 8, the clustering method selected as “pam” (Partitioning Around Medoids), and the distance function nominated as “manhattan,” a consensus clustering analysis was performed using the TCD profile as input. The result of this analysis revealed the identification of four distinct subtypes associated with TCD.

### 2.6 Survival Analysis

To assess the difference in survival among the four identified subtypes of T cell dysfunction, Kaplan–Meier curves were utilized. The survival analysis was performed using the survival package and the survminer package in R.

### 2.7 The Identification of the Tumour Microenvironment Characteristic of 4 Subtypes

The study employed the TIMER [19] algorithm to calculate the median infiltration levels of each immune cell type within the four distinct subtypes of TCD. Using the T‐high subtype as a reference, proportional distributions of immune cell infiltration among the subtypes were calculated. Subsequently, correlation diagrams depicting the interrelationships among immune cell infiltration patterns across the subtypes were generated using the ggcorrplot package. Density plots were utilized to illustrate variations in immune cell infiltration levels among the subtypes, while radar plots were employed to visually represent these variations.

Furthermore, the study conducted computations of the mean expression levels of immune checkpoint genes and T cell inflammation genes within the four subtypes of TCD. This analysis provided insights into the differential expression patterns of these genes across the subtypes.

### 2.8 Analyse of the Cell States Difference Among TCD Subtypes

To characterize the cell states associated with patients suffering from TCD, we obtained 14 cellular states and their corresponding feature genes from CancerSEA. To evaluate the patients' tumour microenvironment (TME), we defined a cellular state score and utilized the GSVA package in R to compute these scores.

The patients were divided into training (60%), test (20%), and validation (20%) datasets for the analysis. Using the randomForest package in R, a classifier was created to predict the T‐high status. The classifier was trained on the training dataset using average gene expression levels associated with invasion and epithelial‐mesenchymal transition (EMT). Following the training phase, the performance of the classifier was assessed using the test and validation datasets. To visualize the performance of the classifier, ROC curves for these datasets were plotted using the pROC package in R.

### 2.9 Identifying Differentially Expressed miRNAs Among the Four Subtypes

The limma package in R was used to identify differentially expressed miRNAs (|log2FC| > 1 and *p* < 0.01), resulting in the selection of six miRNAs. Co‐expression relationships among these six miRNAs based on Pearson correlation scores and their expression levels were described. Patients were stratified into training (70%) and test (30%) datasets. Using the expression levels of the six miRNAs (miR‐203b, miR‐214, miR‐4772 for T‐high subtype; miR‐141, miR‐200a, miR‐200b for T‐low subtype), two classifiers predicting the degree of TCD were constructed in the training dataset using the randomForest method. Subsequently, ROC curves for the test dataset were generated using the pROC package in R. Additionally, patient classification groups were evaluated using Kaplan–Meier survival curves with the survival package in R.

### 2.10 Construction and Validation of miRNAs Prognostic Model

Univariate cox regression analyses were separately performed on the expression of six miRNAs. Multivariate cox regression analysis of five significant prognostic miRNA was performed to obtain the hazard ratio (HR) value for each miRNA. A prognostic model was constructed using expression of five miRNAs. The intensity process ${\hat{h}}_{i}\left(t\right)$ can be written as.

(2) $${\hat{h}}_{i}\left(t\right)={\hat{h}}_{0}\left(t\right)\exp\left(x_{i}^{\prime }\hat{\beta }\right)$$

where ${\hat{h}}_{0}\left(t\right)$ is the baseline hazard function, *β* is the vector of the regression coefficients and $x_{i}^{\prime }\hat{\beta }$ is the risk score for individual *i*. We define *f (X)* = $x_{i}^{\prime }\hat{\beta }$ to be the linear risk score function.

The risk scores were predicted using the score function, and the forestplot package was used to visualize the HR values of the six miRNAs. Based on the predicted risk scores from the prognostic model, patients were divided into high‐risk and low‐risk groups, and a Kaplan–Meier curve was generated using the survminer package. To further validate the impact of these five miRNAs on patient survival, the expression data of the five miRNAs from the GSE92928 and GSE73581 datasets were used. Using the risk scores predicted by the prognostic model, patients in each dataset were separately classified into high‐risk and low‐risk groups, and Kaplan–Meier curves were plotted accordingly.

### 2.11 Functional Enrichment Analysis of miRNA Target Genes

The miRCarta database was used to obtain target genes for the five miRNAs. DEGs were identified using gene expression profiling data and the limma package in R. KEGG enrichment pathway results for the target genes of the five miRNAs were obtained using DAVID. Common and specific genes and pathways among the five miRNAs were counted, and a Sankey diagram illustrating the correspondence between the miRNAs, their target genes, and pathways was created using the ggplot2 package in R. Pearson correlation analysis was performed to assess the relationship between the target genes of the five miRNAs and miRNA expression levels. Genes that exhibited a negative correlation with miRNA expression and higher expression in the T‐low subgroup compared to the T‐high subgroup were selected. A gene network of shared target genes for the five miRNAs was constructed using Pearson correlation analysis. Additionally, the clusterprofiler package was used to draw an enrichment network diagram for the target genes of the miRNAs, with line widths representing the correlation scores.

## 3 Results

### 3.1 Distinct Patterns of T Cell Dysfunction Across Pan‐Cancer Cohorts

Despite the infiltration of cytotoxic T cells (CTLs) into the tumour microenvironment, tumour progression persists in some patients due to TCD. TCD, a key barrier to effective antitumor immunity, significantly influences the outcomes of cancer immunotherapy. To elucidate the mechanisms and patterns of TCD across cancers, we constructed a comprehensive TCD landscape using the expression profiles of 410 genes linked to four distinct TCD states, analysed across 10,363 tumour samples from the TCGA database.

**FIGURE 1:**
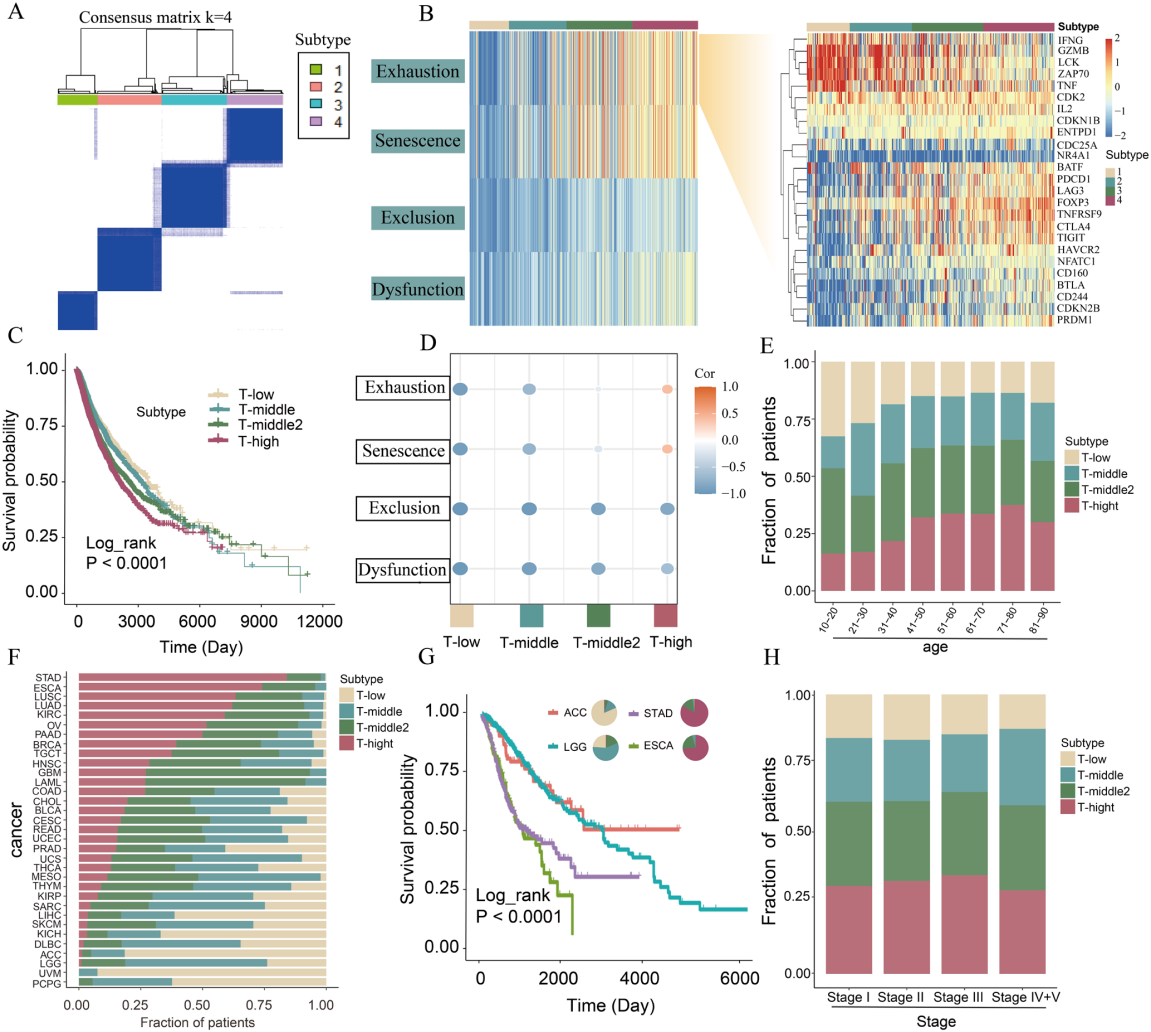
Identification and survival analysis of TCD subtypes. (A) The clustering results of four subtypes when k = 4. (B) Expression differences of the four TCD states and exhaustion genes. Higher expression is represented by colours closer to red. (C) Kaplan–Meier curves of overall survival (OS) among the four TCD subtypes. (D) Expression changes of four TCD states among four subtypes. (E) Differences of patients' age among four TCD subtypes. (F) Differences of cancer types among four TCD subtypes. (G) Survival curves of patients in ACC, ESCA, LGG and STAD. (H) Differences of tumour stages among four TCD subtypes.

Through consensus clustering analysis, we identified distinct TCD patterns in pan‐cancer. The delta area plot of the consensus matrix indicated that k = 4 was the optimal number of clusters, delineating four cancer subgroups (Figure 1A and Figure S1). The consensus matrix heatmap revealed sharp boundaries among these clusters, supporting robust cluster stability (Figure 1A). Based on the degree of TCD, these clusters were labelled as T‐low (subtype 1, *n* = 1802), T‐middle (subtype 2, *n* = 2596), T‐middle2 (subtype 3, *n* = 3002), and T‐high (subtype 4, *n* = 2963), where T‐low represents minimal dysfunction and T‐high reflects maximal dysfunction (Figure 1B and Figure S2). This classification not only highlights four well‐defined cancer clusters but also provides a novel stratification method to evaluate TCD severity across cancer types.

Notably, most genes associated with T cell exhaustion showed a progressive increase in expression as TCD severity rose, underscoring the multifaceted nature of TCD revealed by the analysis of 410 TCD‐related genes (Figure 1B, right). Survival analysis further demonstrated the impact of TCD on patient outcomes. Patients in the T‐low subtype exhibited the most favourable survival rates, whereas those in the T‐high subtype had the poorest prognosis (Figure 1C).

These findings suggest that lower TCD levels are associated with improved survival outcomes, while higher dysfunction levels correlate with worse prognosis. A stepwise increase in TCD severity, particularly in exhaustion and senescence states, was observed from the T‐low to T‐high subtypes, highlighting the prognostic importance of TCD (Figure 1D).

To investigate the clinical features of patients with varying TCD levels, we analysed the distinctive phenotypes of the four TCD subtypes using clinical data from TCGA. Age distribution analysis revealed that younger patients (aged 0 to 60) predominantly fell into the T‐low subtype, constituting 53.7% of this group (Figure 1E). In contrast, older patients (aged over 60) were significantly represented in the T‐high subtype, accounting for 57.6% of this group (Figure 1E). This suggests a correlation between age and TCD severity, with younger patients exhibiting lower levels of TCD and older patients experiencing higher levels.

Further analysis revealed a relationship between the proportion of the T‐high subtype and survival outcomes across cancer types (Figure 1G). Patients with higher T‐high subtype proportions, such as those with STAD and ESCA, exhibited worse survival rates (Figure 1F). Conversely, cancers with lower T‐high proportions, such as ACC and LGG, demonstrated better survival outcomes. These findings suggest that a higher degree of TCD correlates with poorer prognosis. Moreover, we observed no significant variation in the distribution of the four TCD subtypes across different cancer stages (Figure 1H), suggesting that TCD is independent of cancer progression stage. This underscores the critical role of T cell functionality as a distinct factor influencing cancer prognosis.

In conclusion, the clinical characteristics of patients with different degrees of TCD were investigated, revealing distinct age distributions and associations with specific cancer types. These findings highlight the importance of understanding TCD in cancer patients.

### 3.2 Patients With Severe TCD Exhibit Inflammatory TME and Immune Checkpoint Upregulation

**FIGURE 2:**
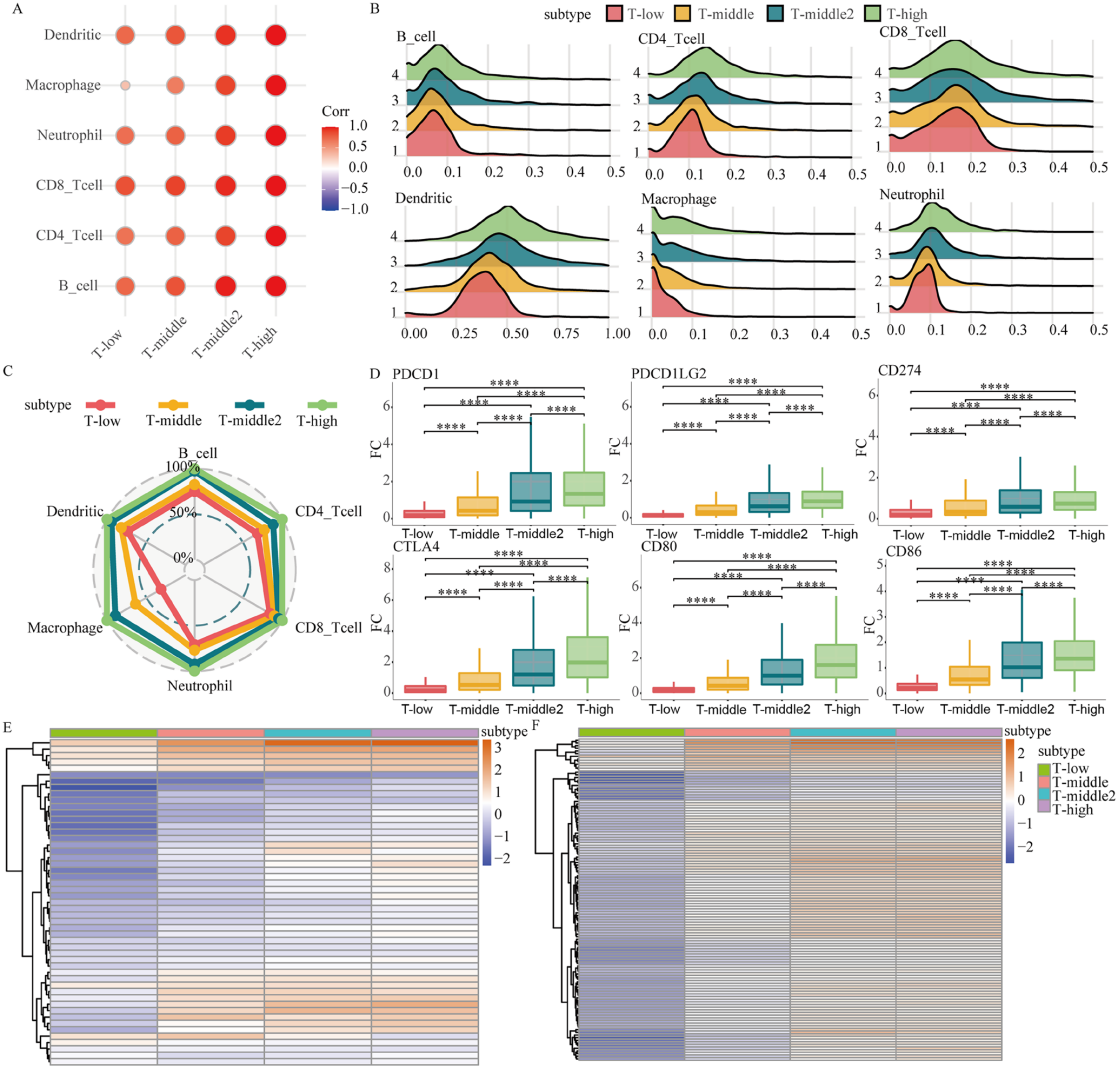
Differences of four TCD subtypes in tumour immune microenvironment. (ABC) Differences in the degree and density of immune cell infiltration among the four TCD subtypes. (D) Differential gene expression of immune checkpoints among four TCD subtypes. (E) Significance test of the expression differences of six immune checkpoint genes among four TCD subtypes. (F) Expression heatmap of T cell inflammation related genes among four TCD subtypes.

TCD is a critical determinant of cancer progression, with higher degrees of dysfunction correlating with poorer patient prognosis. To investigate how TCD shapes the tumour microenvironment (TME) and influences therapeutic outcomes, we analysed immune cell infiltration, immune checkpoint gene expression, and inflammation‐related genes across TCD subtypes (T‐low to T‐high). Using immune cell infiltration data from the TIMER database, we observed a progressive increase in immune cell infiltration, particularly macrophages, from T‐low to T‐high subtypes (Figure 2A). T‐high subtypes displayed distinct macrophage infiltration density distributions, characterized by a left‐skewed peak, indicating increased macrophage presence compared to T‐low subtypes (Figure 2B,C). These results highlight a strong association between severe TCD and elevated macrophage infiltration.

The increased macrophage infiltration observed in T‐high subtypes may suggest a potential contribution of these cells to the upregulation of immune checkpoint genes. To further assess the implications of TCD on immunotherapy responsiveness, we analysed the expression of immune checkpoint‐related genes. Genes such as PDCD1, CTLA4, PDCD1LG2, CD80, CD274, and CD86 were significantly upregulated in T‐high subtypes and downregulated in T‐low subtypes (Figure 2D). This pattern suggests that patients with severe TCD may respond favourably to targeted immunotherapy. Predictors of immunotherapy response, including these immune checkpoint genes, were markedly upregulated in T‐high subtypes (Figure 2E), reinforcing their potential as therapeutic targets. Additionally, inflammation‐related genes were significantly upregulated in T‐high subtypes and downregulated in T‐low subtypes (Figure 2F), indicating that elevated expression of these genes contributes to an inflammatory TME and further promotes T cell dysfunction.

Collectively, our findings indicate that higher degrees of T cell dysfunction are associated with an inflammatory TME characterized by increased macrophage infiltration and upregulated immune checkpoint genes. Tumours are often categorized into two groups based on their immune microenvironment, including hot tumours and cold tumours. Hot tumours are characterized by abundant immune cell infiltration, particularly cytotoxic T lymphocytes (CTLs), indicating an active immune response. However, these tumours may also exhibit immune suppression through upregulation of immune checkpoints, leading to a state of immune exhaustion and reduced effectiveness of immune responses. Cold tumours, on the other hand, are defined by low levels of immune cell infiltration and poor immune activation. These tumours evade immune detection and are less responsive to immune surveillance.

Within the hot versus cold tumour framework, the T‐high subtype is best characterized as immune‐inflamed (hot) but dysfunctional: it displays abundant immune infiltration and inflammatory signals, yet is accompanied by marked immune checkpoint upregulation, consistent with an exhausted and immunosuppressed state rather than productive antitumor immunity. This contrasts with immune cold tumours, where low infiltration is the dominant feature. Notably, macrophage enrichment in T‐high suggests that myeloid populations may help establish an immunosuppressive niche that promotes T cell dysfunction and potentially contributes to T cell exclusion or exhaustion rather than tumour clearance.

### 3.3 Invasion and EMT as Key Drivers of TCD Progression

**FIGURE 3:**
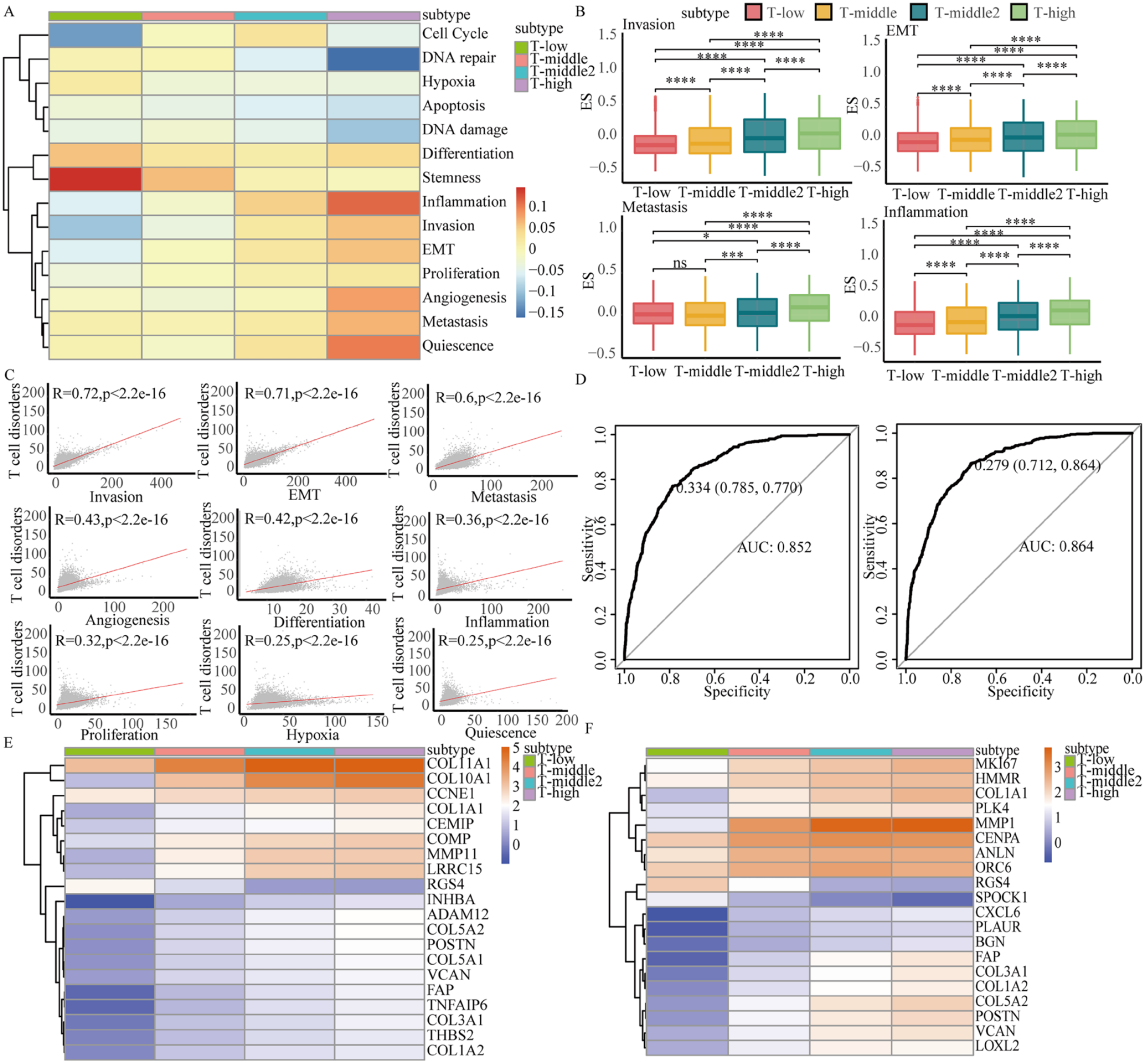
Differences of four TCD subtypes in 14 cell states. (A) Differences in enrichment scores of the 14 cell states among the four TCD subtypes. (B) Significantly test the differences of enrichment scores with four cell states among four TCD subtypes. (C) Correlation analysis of four cell state genes and TCD gene expression. (D) Receiver Operating Characteristic (ROC) curves of the test dataset and validation dataset for the classifier constructed using invasion and EMT gene expression. (E) The expression levels of invasion‐related genes among the four TCD subtypes. (F) The expression levels of EMT‐related genes among four TCD subtypes.

Cell states, reflecting key interactions between the tumour microenvironment and the immune system, are critical drivers of TCD progression. To investigate the relationship between cell states and TCD severity, we calculated enrichment scores for 14 predefined cell states using the GSVA package in R based on the expression levels of their marker genes. Comparison of these scores across the four TCD subtypes (T‐low, T‐middle, T‐middle2, and T‐high) revealed distinct trends. Specifically, inflammation, invasion, EMT, angiogenesis, proliferation, metastasis, and quiescence progressively increased from T‐low to T‐high, whereas apoptosis, DNA damage, DNA repair, hypoxia, and stemness consistently declined (Figure 3A). These results underscore a strong association between cell states and TCD severity. Subgroup‐specific profiles further illustrated unique cell state characteristics within the TCD subtypes. T‐low was characterized by reduced activity in cell cycle, invasion, inflammation, and EMT, alongside heightened activity in stemness, differentiation, hypoxia, and DNA repair. In contrast, T‐high demonstrated reduced activity in apoptosis, DNA repair, DNA damage, and hypoxia, but heightened activity in invasion, EMT, inflammation, angiogenesis, metastasis, and quiescence. These patterns underscore the dynamic cellular reprogramming that occurs with increasing TCD severity, suggesting that TCD subtypes are defined by distinct molecular and functional landscapes.

To further refine these observations, we identified eight cellular states, including invasion, EMT, differentiation, inflammation, cell cycle, apoptosis, proliferation, and quiescence, that differed significantly among the subtypes (*p* < 0.05; Figure 3B and Figure S3). This prompted a more detailed exploration of how these cell states are linked to TCD. Pearson correlation analyses revealed that invasion and EMT exhibited the strongest correlations with TCD (*r* = 0.72 and 0.71, respectively; Figure 3C), emphasizing their pivotal role in TCD progression and offering a mechanistic link between changes in cellular states and TCD severity.

To test the potential clinical utility of invasion and EMT‐related genes, we developed a classifier to identify T‐high patients based on the mean expression of these genes. The classifier achieved robust predictive performance, with AUC values of 0.852 and 0.864 in validation and test datasets, respectively (Figure 3D). These findings highlight the potential of invasion and EMT as diagnostic markers for advanced TCD.

Additionally, analysis of the top 20 genes associated with invasion and EMT revealed consistent upregulation from T‐low to T‐high. Notable genes included COL11A1 and INHBA (invasion) and MMP1 and CXCL6 (EMT), which exhibited particularly strong increases in expression (Figure 3E,F). These findings further validate the central role of invasion and EMT in driving TCD progression.

Taken together, our results demonstrate a hierarchical progression of cellular state alterations across TCD subtypes, with invasion and EMT emerging as critical drivers of TCD severity.

### 3.4 Patients With T‐High Subtype Exhibited Low Methylation Levels

**FIGURE 4:**
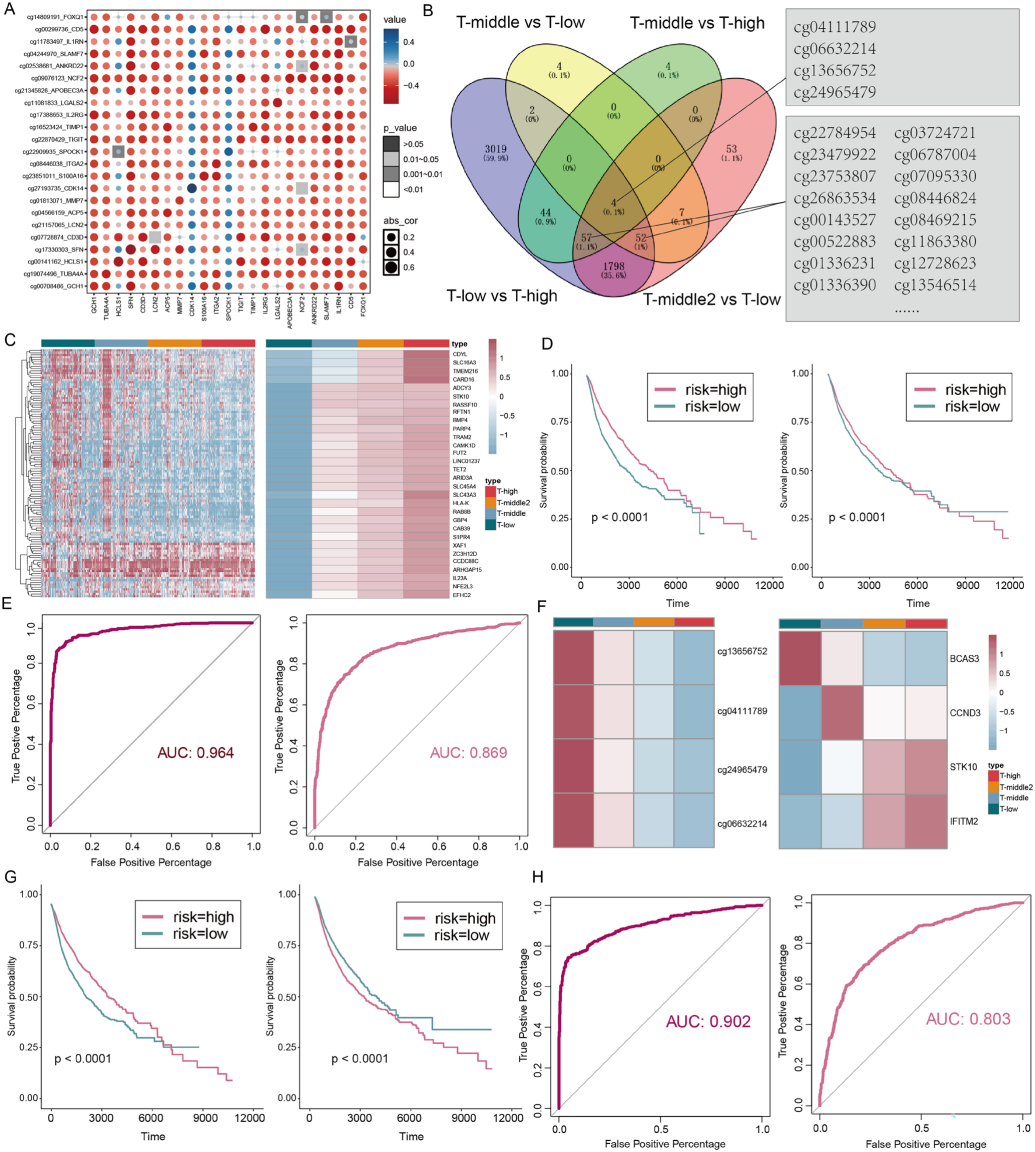
Prognostic model construction of TCD‐related methylation. (A) The correlation analysis of TCD genes and associated methylation. (B) Differential methylation probes among the four TCD subtypes. (C) The distribution of DMPs in the four TCD subtypes was exhibited by heatmap (left), expression of genes corresponding to down‐regulated DMPs in the four TCD subtypes (right). (D) Survival curves of the prognostic model were constructed based on the expression of DMPs (left) and genes corresponding to downregulated DMP (right). (F) The four DMPs consistently decrease from the T‐low subtype to the T‐high subtype (left), expression heatmap of STK10 and IFTM2 genes. (H) The classifier ROC curves constructed from the four DMPs (left) and two genes (right).

DNA methylation, a key epigenetic modification, regulates gene expression and influences TCD, contributing to immune suppression and its progression. To investigate the role of DNA methylation in influencing TCD, we explored the relationship between TCD and DNA methylation to uncover its epigenetic mechanisms. We investigated the relationship between TCD and DNA methylation, and correlation analysis using 450 k DNA methylation data from UCSC Xena revealed a significant negative correlation between methylation levels and TCD‐related gene expression, suggesting that hypomethylation activates key genes associated with TCD (Figure 4A). Differentially methylated probe (DMP) analysis across the four TCD subtypes identified 109 DMPs (Δbeta > 0.15, FDR < 0.05), revealing a progressive decline in methylation levels from the T‐low to the T‐high subtype, accompanied by a corresponding increase in the expression of DMP‐associated genes (Figure 4B,C). These findings indicate that hypomethylation enhances gene activity, contributing to TCD severity.

To assess the clinical relevance of these features, univariate and multivariate Cox proportional hazard regression models identified key prognostic DMPs and genes associated with overall survival. Kaplan–Meier survival analysis showed that patients in the high‐risk group, defined by lower methylation or higher gene expression levels, had significantly poorer survival compared to the low‐risk group (Log‐rank test, *p* < 0.001, Figure 4D). Classifiers constructed using these features achieved high predictive accuracy for TCD severity, with AUC values of 0.964 for methylation and 0.869 for gene expression (Figure 4E).

In order to more accurately identify prognostic features associated with TCD. We analysed the four DMPs (cg04111789, cg06632214, cg13656752, cg24965479) in intersecting among the four groups of DMPs (Figure 4B). The results revealed that the methylation levels of the four DMPs consistently decrease from the T‐low subtype to the T‐high subtype (Figure 4F, left). Simultaneously, we observed a gradual increase in the expression of the DMP‐related genes (STK10, IFTM2) from the T‐low subtype to the T‐high subtype (Figure 4F, right).

Therefore, the DMPs (cg04111789, cg06632214, cg13656752, cg24965479) were used to establish a multiple Cox proportional hazards regression model and the Kaplan–Meier survival analysis showed that the high‐risk group had poor overall survival compared with the low‐risk group (Log‐rank test *p* < 0.001, Figure 4G, left). Similarly, genes STK10 and IFTM2 were used to establish a multivariate Cox proportional hazards regression model. Kaplan–Meier survival analysis showed a trend consistent with the survival results of the DNA methylation model associated with TCD (Log‐rank test *p* < 0.001, Figure 4G, right). In addition, we constructed classifiers based on the levels of these prognosis‐related methylation (AUC, 0.902, Figure 4H, left) and gene expression (AUC, 0.803, Figure 4E, right), using random forest methods to predict the degree of TCD in patients. The results suggested that these prognostic features classify patients with TCD.

Together, the above results demonstrate that T‐high subtype with hypomethylation levels and the identification of TCD related prognostic signatures from DNA methylation and gene expression alterations accurately predicts patients' risk.

However, to further deepen our understanding of T cell dysfunction, it is essential to investigate the role of miRNA profiles and their potential contribution to the predictive models for patient risk assessment.

### 3.5 miRNAs as Predictive Biomarkers for T Cell Dysfunction Sseverity

**FIGURE 5:**
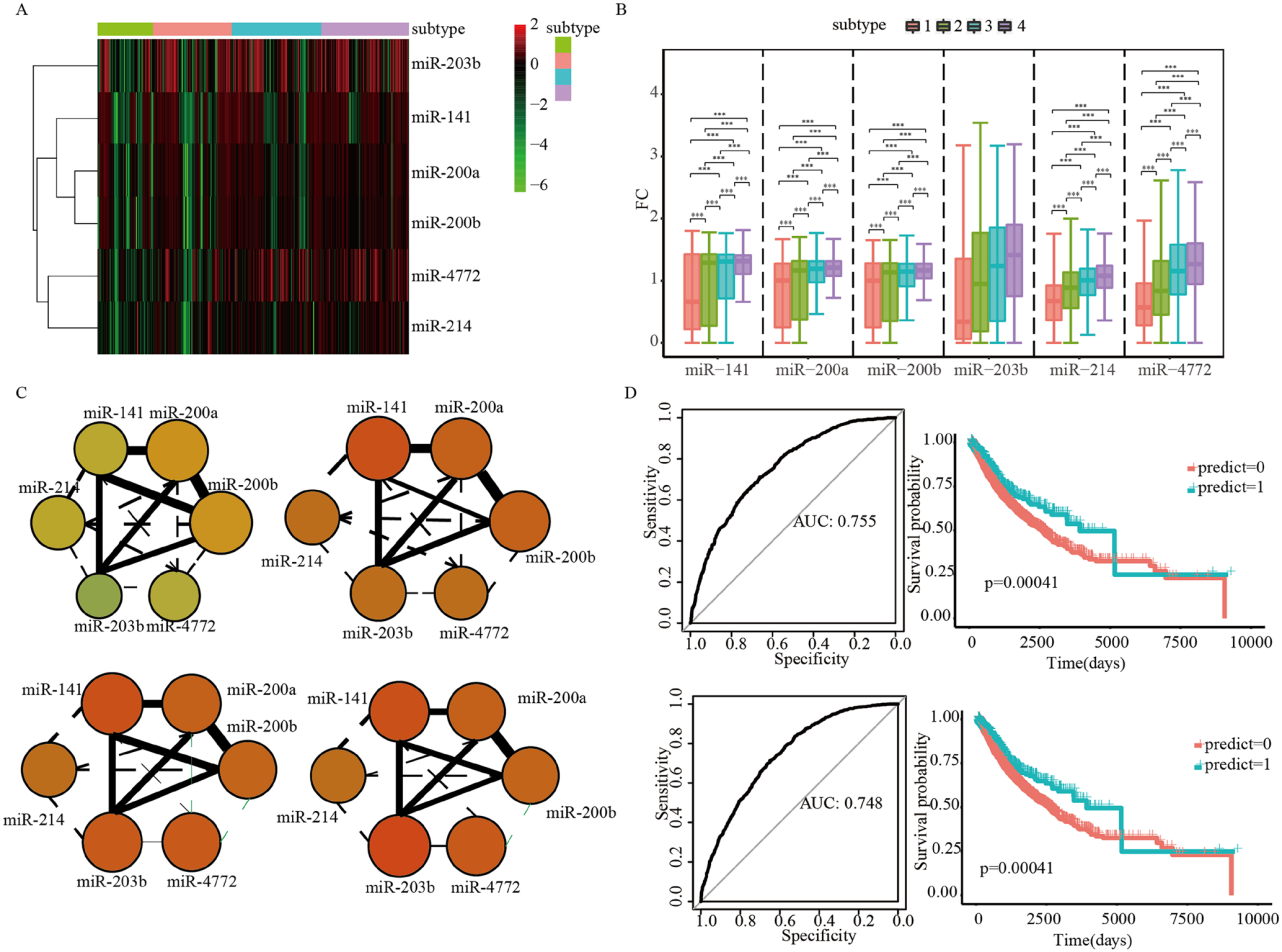
Identification of a differentially expressed miRNA in the four TCD subtypes. (A) Expression levels of six differentially expressed miRNA among four TCD subtypes. (B) Significance test of the six differentially expressed miRNA in the four TCD subtypes. (C) Correlation analyses of the six differentially expressed miRNAs among the four TCD subtypes. The colour closer to yellow indicates higher expression, and the thickness of the line represents the strength of correlation. (D) ROC curves of two classifiers constructed by miRNA expression data and survival curves of two test datasets. The classifier above was constructed using miR‐141, miR‐200a, and miR‐200b, and the classifier below was constructed using miR‐203b, miR‐214, and miR‐4772.

MiRNAs had been implicated in TCD and had the potential to serve as biomarkers. We investigated the potential of miRNAs as biomarkers for TCD subtypes. We investigated the potential of miRNAs as biomarkers for TCD subtypes. We identified six miRNAs (miR‐203b, miR‐141, miR‐200a, miR‐200b, miR‐4772, and miR‐214) that were differentially expressed across four TCD subtypes, with expression levels progressively increasing from T‐low to T‐high subtypes (*p* < 0.05; Figure 5A,B). These findings suggest that the expression and regulatory capacity of these miRNAs are enhanced with increasing TCD severity.

Pearson correlation analyses further revealed significant relationships among the differentially expressed miRNAs (Figure S4). Interestingly, as miRNA expression levels increased with TCD severity, correlation values between miRNAs decreased, indicating potential functional divergence. Notably, miR‐200a and miR‐200b showed the highest correlation, while miR‐4772 and miR‐200a exhibited the lowest correlation (Figure 5C). Among these, miR‐141, miR‐200a, miR‐200b, and miR‐203b consistently exhibited strong associations across all TCD subtypes, highlighting their potential as robust biomarkers for TCD.

To evaluate the predictive value of these miRNAs, we constructed classifiers for T‐low and T‐high subtypes using specific miRNA combinations. For the T‐low subtype, a classifier incorporating miR‐141, miR‐200a, and miR‐200b achieved an area under the curve (AUC) of 0.755, effectively identifying patients with low TCD severity (Figure 5D). Survival analysis demonstrated significant survival differences between the predicted T‐low and non‐T‐low groups. Similarly, a classifier for the T‐high subtype, comprising miR‐203b, miR‐214, and miR‐4772, achieved an AUC of 0.748 (Figure 5D). Predicted T‐high patients exhibited significantly different survival outcomes compared to non‐T‐high patients, underscoring the predictive power of these miRNA combinations.

Collectively, these findings establish a strong association between specific miRNA expression profiles and TCD severity. Furthermore, the predictive capacity of miRNA‐based classifiers supports their utility as potential biomarkers for stratifying TCD subtypes.

### 3.6 Exploring the Potential of miRNAs as Prognostic Markers for T Cell Dysfunction

**FIGURE 6:**
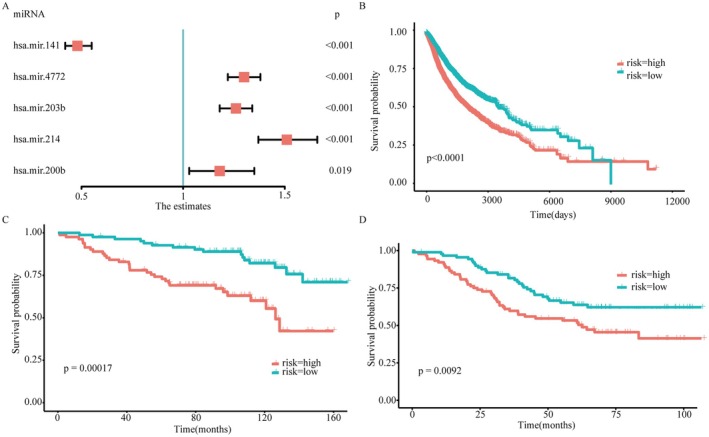
Construction and verification of miRNA prognosis model. (A) Hazard ratio (HR) values obtained through multivariate Cox regression analysis of five miRNAs. (B) Kaplan–Meier curves show the independent relevance between overall survival time and risk scores. (C) Validation of prognosis model using miRNA data from GSE92928. (D) Validation of prognosis model using miRNA data from GSE73581.

The above found that those signature miRNAs effectively predict TCD subtypes and are significantly correlated with patient survival. Therefore, to investigate whether these signature miRNAs can serve as independent prognostic markers for predicting patient survival. We performed a univariate cox regression analysis to identify significant prognostic genes (*p* < 0.05), including miR‐141, miR‐4772, miR‐203b, miR‐214, and miR‐200b, which were further used to establish multivariate Cox proportional risk regression models (Figure 6A). The hazard ratios (HR) were determined for each miRNA, and all miRNAs, except miR‐141, had HR values greater than 1. Moreover, the *p*‐values for all miRNAs were less than 0.05, indicating their significant impact on patient survival and their suitability for constructing a prognosis model. Using the scoring function, we generated a predictive risk score. Based on the risk scores predicted by the prognosis model, the patients were stratified into high‐risk and low‐risk groups. The survival curve analysis revealed a significant difference in patient survival time between the high‐risk and low‐risk groups (*p* < 0.0001, Figure 6B), suggesting that these four signature miRNAs could serve as independent prognostic factors.

Next, to validate the applicability of the prognosis model, we employed independent datasets (GSE92928 and GSE73581) to assess its effectiveness. The prognostic value of the model was confirmed in both the GSE92928 (*p* = 0.00017, Figure 6C) and GSE73581 (*p* = 0.00092, Figure 6D) cohorts, further affirming the effectiveness of the established prognostic model of miRNA expression in accurately predicting patient prognosis.

Overall, the identified signature miRNAs (miR‐141, miR‐4772, miR‐203b, miR‐214, and miR‐200b) proved effective in predicting TCD subtypes and served as independent prognostic markers, providing valuable insights into the molecular mechanisms underlying T cell dysfunction.

### 3.7 Analysis of Signature miRNA Target Genes Reveals Enrichment in TCD and Cancer‐Related Pathways

The above results found that miRNA associated with TCD can be used as a prognostic marker and can serve as a biomarker to predict the degree of T cell dysfunction in patients, implying that these miRNAs are tightly associated with TCD; therefore, we then analysed the function of the above characterized miRNA target genes.

**FIGURE 7:**
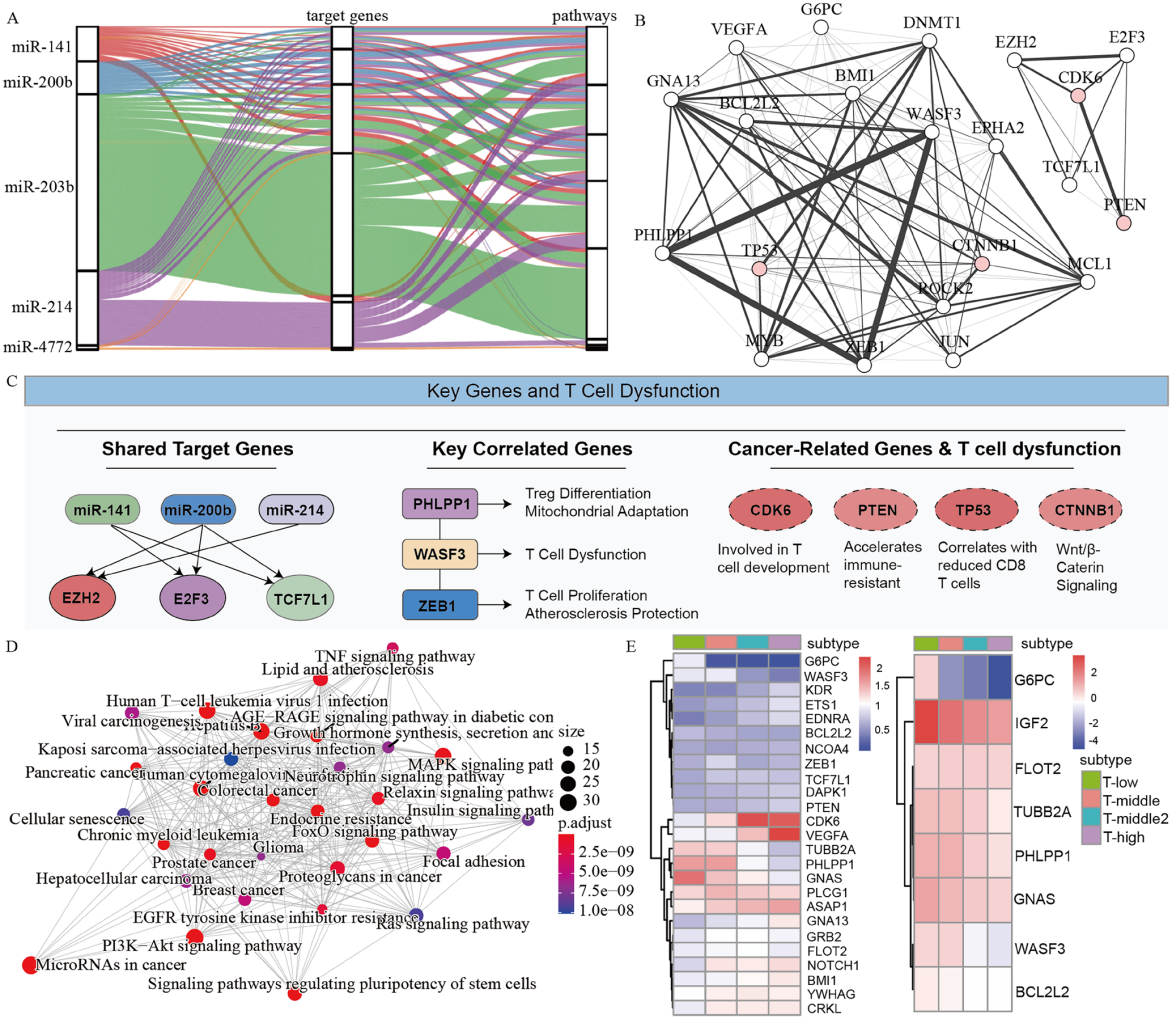
Functional enrichment analysis of miRNA target genes. (A) Common target genes and pathways shared by five miRNAs. (B) Gene network map of common target genes shared by five miRNAs. The thickness of the line represents the strength of correlation, and the red genes are known cancer‐related genes. (C) Key genes and their mechanisms of T cell dysfunction. (D) Network diagram of functional enrichment analysis results of miRNA target genes. (E) The expression levels of genes negatively associated with the miRNAs, as well as the expression levels of genes with higher expression in the T‐low subtype compared to the T‐high subtype.

Utilizing miRNA target gene data from the miRCarta database, specific target genes and associated pathways for the five miRNAs were identified. A Sankey diagram illustrates shared common differentially expressed target genes and pathways among these miRNAs, with miR‐203b displaying the highest number of differentially expressed target genes related to pathways (Figure 7A; Table S3). This suggests that these miRNAs collectively serve similar biological functions.

To explore the relationships among the common differentially expressed target genes, we computed Pearson correlation coefficients and constructed a target gene network diagram (Figure 7B,C). Within this network, E2H2, E2F3, CDK6, TCF7L1, and PTEN were identified as common targets shared by three miRNAs. Additionally, PHLPP1, WASF3, and ZEB1 exhibited significant correlations. PHLPP1 plays a crucial role in Treg cell differentiation and mitochondrial adaptability through its phosphatase activity [20]. ZEB1 regulates T cell proliferation to prevent atherosclerosis and promotes T cell differentiation [21, 22]. WASF3 may also be involved in TCD. To further validate these target genes, we consulted the miRDB database [23]. Several of the previously identified targets were confirmed as high‐confidence targets of the corresponding miRNAs, with an average target score of approximately 80. For instance, E2F3 and ZEB1 were confirmed targets of hsa‐miR‐141, and ZEB1 and WASF3 were confirmed targets of hsa‐miR‐200b. These results further support the reliability of our identified miRNA‐target interactions (Table S4).

Notably, several known cancer‐related genes, including CDK6, PTEN, TP53, and CTNNB1, were identified within the network. CDK6 promotes leukemia and lymphoma by regulating haematopoietic T cell development [24]. PTENα accelerates immune‐resistant cancer progression and induces TCD [25]. TP53 mutations correlate with decreased CD8+ T cell density and reduced bone marrow infiltrating OX40+ cytotoxic T cells and helper T cells [26, 27, 28]. CTNNB1 mutations confer resistance to immunotherapy by inhibiting T cell differentiation via Wnt/β‐catenin signaling [29]. These findings underscore the close relationship between these miRNAs and TCD (Figure 7B,C).

Further analysis revealed an interconnected network of pathways involving miRNA regulation, T cell dysfunction, and cancer progression (Figure 7D), indicating that altered miRNA expression influences both T cell dysfunction and cancer‐related pathways through the regulation of target gene expression.

Moreover, through Pearson correlation analysis, several genes were identified that showed a negative correlation with miRNA expression and exhibited higher expression levels in T‐low compared to T‐high (Figure 7E). These genes include G6PC, IGF2, FLOT2, TUBB2A, PHLPP1, GNAS, WASF3, and BCL2L2, and they are closely associated with miRNA regulation. Notably, PHLPP1 is linked to PTEN and plays a role in inducing Treg cell differentiation [20], while FLOT2 is involved in transcriptional regulation, cell death, and neuroinflammation [30]. These findings highlight the intricate relationships between these genes and miRNA expression levels, emphasizing their impact on TCD.

In summary, the analysis indicates that miRNA target genes are enriched in pathways related to T cell dysfunction and cancer, illustrating that changes in miRNA target gene expression influence both T cell function and cancer development.

## 4 Discussion

T cells play a pivotal role in the immune response against tumours, and their dysfunction significantly impairs anti‐tumour immunity. Investigating the aetiology and pathogenesis of T cell dysfunction (TCD) is critical for understanding tumour immune evasion and improving therapeutic strategies [31]. In this study, we constructed a comprehensive pan‐cancer landscape of TCD, identifying four subtypes, T‐low, T‐middle, T‐middle2, and T‐high which reflect a progressive deterioration of T cell function. This classification not only provides a deeper understanding of TCD but also highlights its clinical relevance across multiple cancer types.

A key finding of our study is the identification of six miRNAs, miR‐203b, miR‐214, miR‐4772, miR‐141, miR‐200a, and miR‐200b, which serve as reliable biomarkers for predicting the severity of TCD. These miRNAs exhibited distinct expression patterns across the four TCD subtypes, with higher expression levels correlating to increased TCD severity and poorer patient outcomes. Importantly, miRNA‐based classifiers demonstrated strong predictive performance, enabling the stratification of patients into high‐risk and low‐risk groups. This provides a promising tool for improving risk assessment and guiding personalized cancer therapy.

The functional enrichment analysis of target genes regulated by these miRNAs revealed their involvement in pathways related to T cell dysfunction and cancer progression, including immune checkpoint regulation, invasion, and epithelial‐mesenchymal transition (EMT). Specifically, invasion and EMT were found to be strongly correlated with TCD severity, indicating that tumour microenvironmental changes may exacerbate T cell dysfunction through molecular reprogramming. These findings suggest a dual role of the identified miRNAs: as both regulators of TCD and contributors to broader oncogenic processes.

In addition to miRNA biomarkers, our analysis of DNA methylation patterns revealed that patients with severe TCD (T‐high subtype) exhibited low methylation levels. Dysregulated methylation likely contributes to aberrant gene expression that promotes T cell exhaustion and senescence. Importantly, prognostic models based on DNA methylation and TCD‐related gene expression further demonstrated their utility in risk prediction, reinforcing the relevance of epigenetic regulation in T cell dysfunction. Our observation that DNA methylation levels are inversely associated with TCD‐related gene expression suggests that epigenetic dysregulation may contribute to the establishment and maintenance of severe dysfunction. This raises the possibility of epigenetic priming pharmacologic epigenetic modulators could potentially reprogram immune‐related transcriptional programs in tumour and stromal compartments and may be explored in combination with immune checkpoint blockade to enhance immunotherapy responsiveness. However, since the T‐high subtype in our dataset already exhibits globally reduced methylation, it will be important to determine whether therapeutic benefit might arise from context‐ and locus‐specific reprogramming rather than further global demethylation. Future experimental and clinical studies are needed to evaluate whether epigenetic intervention can shift T‐high tumours toward a less dysfunctional and more immunotherapy‐responsive state.

Despite the presence of significant immune cell infiltration in the T‐high subtype, our study revealed that many of these immune cells remain in a dysfunctional state, unable to mount effective anti‐tumour responses. This highlights the complexity of the tumour immune microenvironment (TME), where increased immune infiltration may paradoxically coexist with immunosuppression. Elevated expression of immune checkpoint genes (e.g., PDCD1, CTLA4) in the T‐high subtype suggests that patients with severe TCD might benefit from immune checkpoint blockade therapies, providing a potential avenue for therapeutic intervention.

Age is a major determinant of immune competence. The enrichment of patients over 60 years old in the T‐high subtype suggests that immunosenescence and inflammaging may lower the baseline threshold for T cell dysfunction, thereby predisposing older patients to more severe dysfunctional states. Nevertheless, the T‐high tumours also exhibit hallmark features of tumour microenvironment driven dysfunction, implying that tumour‐derived suppressive cues may further exacerbate exhaustion on top of baseline immune aging. Because TCGA does not provide pre‐cancer immune profiling or longitudinal immune‐age metrics, we cannot fully distinguish tumour‐induced dysfunction from baseline immune aging, and future prospective studies with matched peripheral immune phenotyping will be required. These findings underscore the importance of considering immune age when stratifying older patients for immunotherapy and evaluating rational combination strategies to overcome severe dysfunction in the elderly.

In conclusion, our study offers a comprehensive portrayal of TCD across cancers and identifies six miRNA biomarkers (miR‐203b, miR‐214, miR‐4772, miR‐141, miR‐200a, and miR‐200b) as critical regulators and predictors of TCD severity. These miRNAs, together with DNA methylation patterns and molecular classifiers, provide robust tools for patient stratification, prognostic evaluation, and therapeutic exploration. By integrating multi‐omics data, we not only enhance our understanding of the molecular mechanisms underlying TCD but also pave the way for personalized treatment strategies aimed at overcoming T cell dysfunction in cancer.

## Author Contributions

**Zhe‐yu Wu:** methodology, validation, visualization. **Fei‐fan Xiong:** resources. **Jiang‐ying Liang:** resources. **Deng‐hui Guo:** resources. **Hong‐jiu Wang:** conceptualization, methodology, formal analysis, writing – original draft. **Na Wang:** conceptualization, supervision. **Si‐rui Li:** resources. **Jie Shen:** resources. **Shu‐heng Fu:** resources. **Xiao‐ling Wen:** methodology validation formal analysis, writing – original draft preparation. **Zhen‐zhen Wang:** conceptualization, writing – review and editing, supervision. **Xiao‐ling Gao:** conceptualization, writing – review and editing.

## Funding

This work was supported by Natural Science Foundation of Hainan Province, No. 822MS074, No. 821MS0777, No. 821MS045, No. 621MS041. National Natural Science Foundation of China, No. 32160179, No. 31701159, No. 32500823. Education Department of Hainan Province, No. Hnky2022‐32, No. Hnky2025‐24. Key Research and Development Project of Hainan Province, No. ZDYF2022SHFZ055. Hainan Province Clinical, Medical Center.

## Conflicts of Interest

The authors declare no conflicts of interest.

## Supporting information


**Figure S1:** A cumulative distribution function (CDF) curve with k = 2–8.

**Figure S2:** Gene expression heatmap of TCD states. (A) The expression heatmap of exclusion related genes. (B) The expression heatmap of senescence related genes. (C) The expression heatmap of dysfunction related genes.

**Figure S3:** Significantly test the differences of enrichment scores with four cell states among four TCD subtypes.

**Figure S4:** Correlation analysis of expression between miRNA pairwise among four TCD subtypes.

**Table S1:** The immune checkpoint‐related genes.

**Table S2:** The T cell inflammation‐associated genes.

**Table S3:** The miRNA target genes and associated pathways.

**Table S4:** Predicted miRNA‐target gene interactions assessed using miRDB database.

## Data Availability

All data used in this study were obtained from publicly available datasets. The data that support the findings of this study are available from the corresponding author upon reasonable request.
