# Supplementary figures and images for: Novel Prognostic and Predictive miRNA Biomarkers Shape the Landscape of T Cell Dysfunction in Cancer
Source: J Cell Mol Med. 2026 Apr 2;30(7):e71117. doi: 10.1111/jcmm.71117 (PMC13045257; doi:10.1111/jcmm.71117)

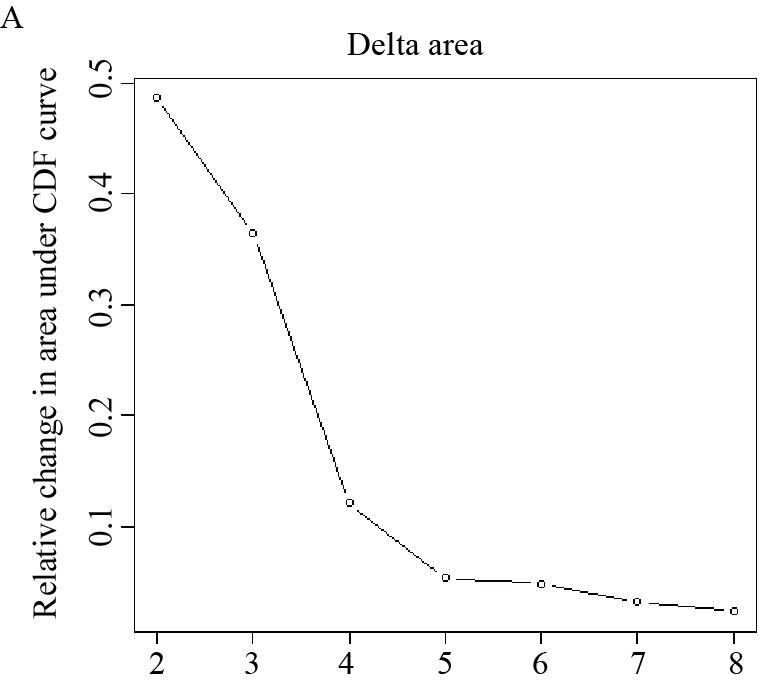

Supplement: Supplementary file 1 — Figure S1: A cumulative distribution function (CDF) curve with k = 2–8. [file JCMM-30-e71117-s001.png]

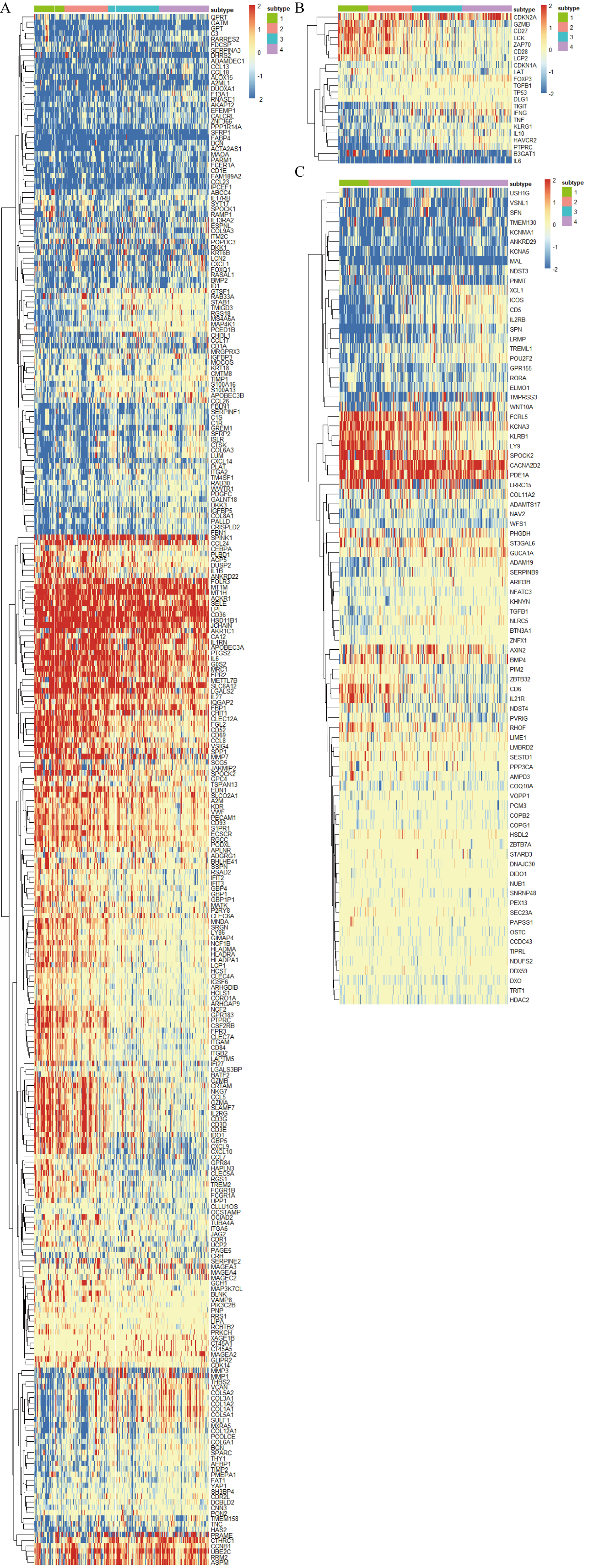

Supplement: Supplementary file 2 — Figure S2: Gene expression heatmap of TCD states. (A) The expression heatmap of exclusion related genes. (B) The expression heatmap of senescence related genes. (C) The expression heatmap of dysfunction related genes. [file JCMM-30-e71117-s005.png]

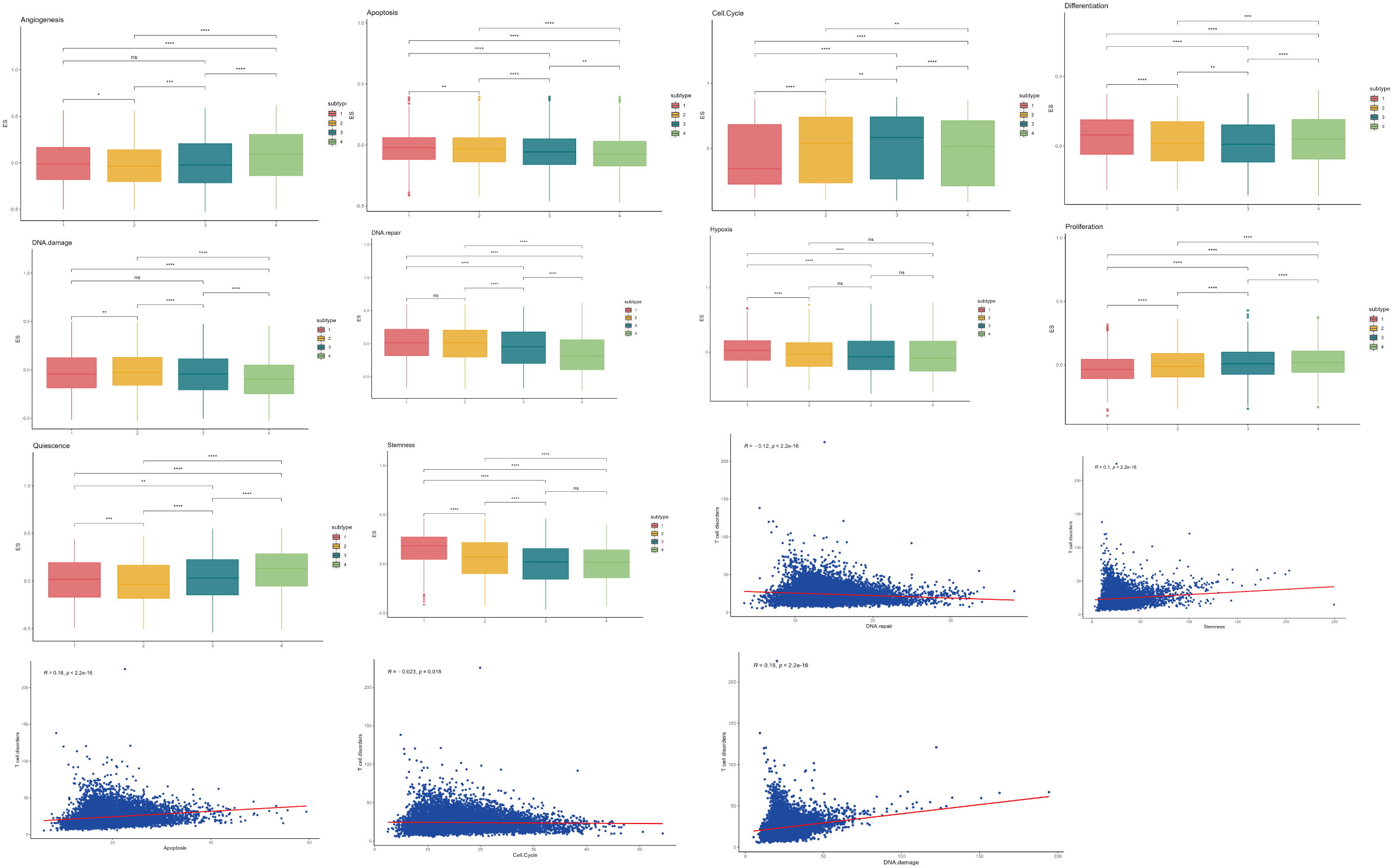

Supplement: Supplementary file 3 — Figure S3: Significantly test the differences of enrichment scores with four cell states among four TCD subtypes. [file JCMM-30-e71117-s008.png]

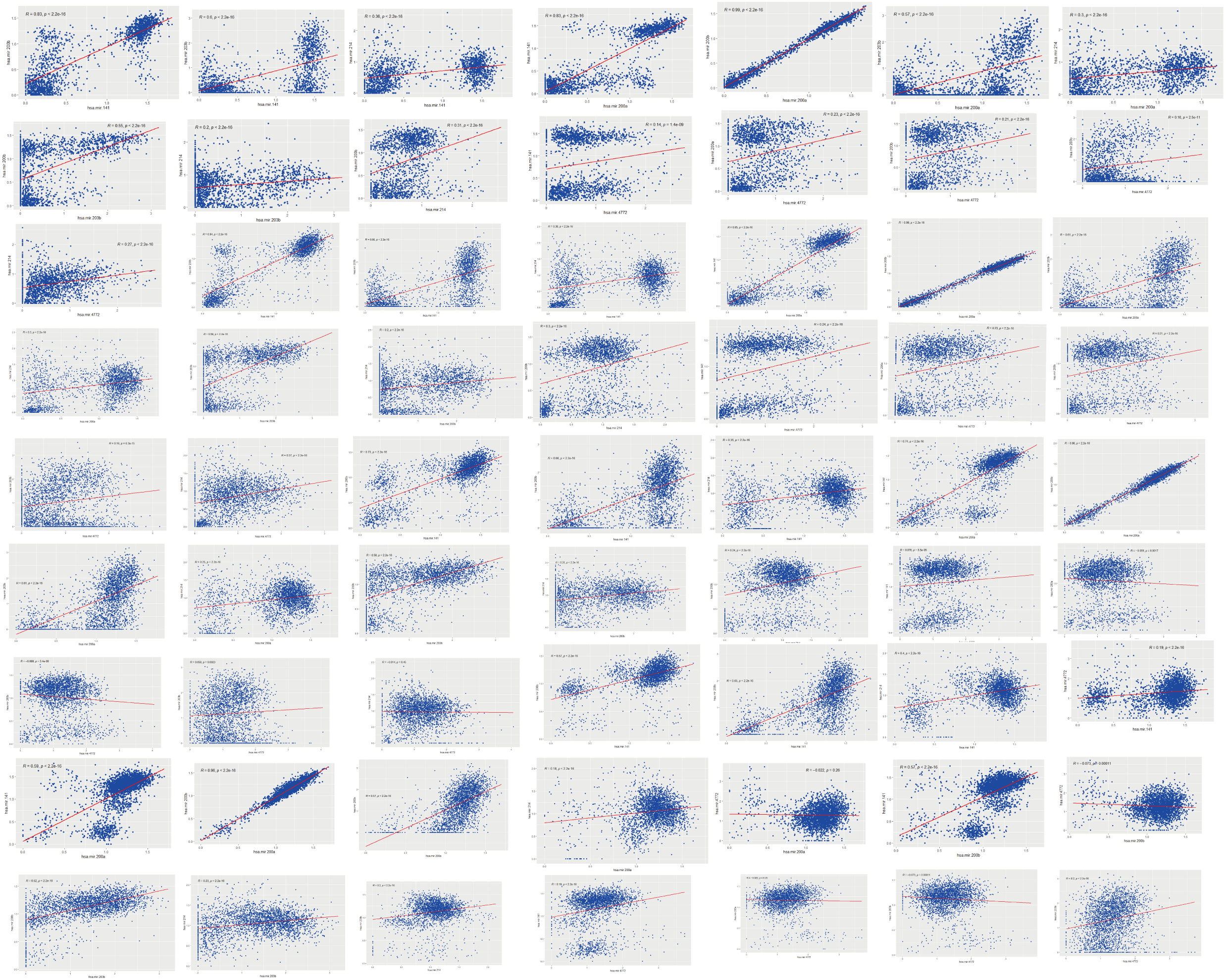

Supplement: Supplementary file 4 — Figure S4: Correlation analysis of expression between miRNA pairwise among four TCD subtypes. [file JCMM-30-e71117-s002.png]
